# Clinical significance of elevated tumor markers in patients with biliary pancreatitis

**DOI:** 10.3389/fmed.2025.1486963

**Published:** 2025-06-04

**Authors:** He Han, Zhiyuan Li, Yunfan Li, Liwen Zhang, Jixiang Chen, Xin Fan

**Affiliations:** ^1^Department of General Surgery, Affiliated Hospital of Jiangsu University, Zhenjiang, Jiangsu, China; ^2^Department of Respiratory, Affiliated Hospital of Jiangsu University, Zhenjiang, Jiangsu, China

**Keywords:** elevated, tumor markers, biliary, pancreatitis, clinical significance

## Abstract

**Objective:**

This study explores the clinical significance of elevated tumor markers in patients with biliary pancreatitis. It aims to develop a machine learning-based clinical prediction model to facilitate early intervention and improve outcomes in acute biliary pancreatitis (ABP).

**Methods:**

We collected data from patients admitted with biliary pancreatitis to the Department of General Surgery at Jiangsu University Hospital from January 1, 2016, to December 31, 2023. We recorded general patient information.

**Results:**

Markers including Carbohydrate Antigen (CA) 50, CA19-9, CA125, CA724, CA242, ferritin, leukocyte count, high-sensitivity C-reactive protein (HS-CRP), total bilirubin, direct bilirubin, alanine aminotransferase, and aspartate aminotransferase were significantly higher in the severe acute pancreatitis (SAP) and moderately severe acute pancreatitis (MSAP) groups compared to the mild acute pancreatitis (MAP) group (*P* < 0.05). Univariate logistic regression analysis identified white blood cell count, HS-CRP, CA50, CA19-9, CA125, urinary amylase, total bilirubin, aspartate aminotransferase, and hospitalization duration as risk factors for progression to MSAP or SAP. Multivariate logistic regression analysis confirmed hospitalization duration as an independent risk factor.

**Conclusion:**

Elevated tumor markers have clinical significance in biliary pancreatitis. We propose a clinical prediction model based on machine learning to screen variables and guide treatment adjustments for MAP.

## 1 Introduction

Acute pancreatitis (AP) is a common surgical acute abdominal condition with an annual incidence of approximately 3.374 per 10,000 individuals and a mortality rate of 0.116 per 10,000 (1, 2). The rising incidence, influenced by lifestyles high in salt, oil, and fat, requires increased attention to this patient population (3). Acute biliary pancreatitis (ABP), the most prevalent form of AP, constitutes a significant proportion of surgical emergency abdominal cases. AP can progress rapidly, triggering both local and systemic inflammatory responses, which increase the risk of multiple organ failure (MOF) and infections. In severe cases, mortality rates can reach up to 30% (4). Approximately 20% of patients with AP progress to moderate or severe disease, experiencing complications such as acute peripancreatic fluid collection, pancreatic pseudocyst, acute necrotizing fluid collection, and wall necrosis (5, 6). These considerations underscore the need for more accurate clinical prediction models for moderate-to-severe pancreatitis.

Several clinical scoring systems are currently used, such as the Acute Physiology and Chronic Health Evaluation II (APACHE-II) score (7), the Bedside Index for Severity in Acute Pancreatitis (BISAP) score (8), and the modified Ranson score (9). However, each system has its limitations. The APACHE-II score requires extensive data for predicting severe acute pancreatitis (SAP), and its predictive value within 24 hours is relatively poor (10, 11); it also has lower specificity than the Ranson score (12). The BISAP score includes subjective assessments of mental status (13), and the Ranson score can only be calculated 48 h after admission, which limits early risk stratification (9). Early clinical observations have shown that many patients with biliary pancreatitis present with significantly abnormal tumor markers upon admission, providing a unique research opportunity (14).

This study explores the clinical significance of these elevated markers in patients with biliary pancreatitis and aims to develop a machine learning-based clinical prediction model to facilitate early intervention and improve the prognosis of patients with ABP.

## 2 Materials and methods

### 2.1 General information

We retrospectively collected clinical data from patients with biliary pancreatitis admitted to the Department of General Surgery at Jiangsu University Hospital between January 1, 2016, and December 31, 2023. Participants were selected based on predefined exclusion and inclusion criteria to ensure a focused and relevant study population. This study was conducted in accordance with the ethical principles of the 1964 Declaration of Helsinki and received approval from the Ethics Committee of Jiangsu University Hospital.

The inclusion criteria were as follows: (1) diagnosed with ABP per the 2021 Acute Pancreatitis Diagnosis and Treatment Guidelines; (2) had gallbladder stones confirmed by computed tomography (CT), magnetic resonance cholangiopancreatography (MRCP), or ultrasound; (3) were aged ≥ 18 years; (4) had no prior history of AP; and (5) underwent complete gastrointestinal tumor markers testing upon admission.

The exclusion criteria were as follows: (1) patients with non-biliary pancreatitis; (2) age ≤ 18 years; (3) patients with multiple prior episodes of AP; (4) patients with concurrent gastrointestinal tract tumors or a history of malignancy; and (5) cases with incomplete clinical data.

According to the revised Atlanta classification (RAC) (15, 16), pancreatitis severity was categorized into mild AP (MAP), moderately severe AP (MSAP), and severe AP (SAP). For the purposes of clinical model construction and analysis, patients categorized as MSAP and SAP were combined into a single non-MAP group, aiming to refine the focus on more severe cases, which are critical for the study’s objectives.

### 2.2 Data collection

General patient information collected at the outset of the study included age, sex, height, hypertension status, and whether the patient had been diagnosed with diabetes mellitus. Upon admission, a series of laboratory tests were performed to establish a comprehensive baseline for each patient. These tests included measurements of the white blood cell count (WBC) and high-sensitivity C-reactive protein (hs-CRP), along with a suite of liver function tests such as total bilirubin, direct bilirubin, alanine aminotransferase, and aspartate aminotransferase. Additionally, a range of tumor markers was assessed, including alpha-fetoprotein (AFP), carcinoembryonic antigen (CEA), and carbohydrate antigens CA50, CA19-9, CA125, CA724, and CA242, as well as ferritin levels. Further data collected encompassed the presence of common bile duct stones, the diagnosis of acute cholecystitis, and the duration of each patient’s hospitalization. Patients were categorized according to the severity of their condition using the revised Atlanta classification at the time of admission, facilitating subsequent analyses based on these initial assessments.

### 2.3 Statistical analyses

Data analysis was conducted using SPSS 26.0 (IBM Corp., Armonk, NY, United States) and GraphPad Prism 9.5.0. Normally distributed continuous variables were expressed as mean ± standard deviation and analyzed with the Kruskal–Wallis test. Categorical variables were presented as percentages (%) and assessed using χ^2^ tests or Fisher’s exact tests, as appropriate. Further advanced statistical modeling was performed using R software (version 3.5.2). For variable selection, the least absolute shrinkage and selection operator (LASSO) regression was employed. This method was chosen because it effectively manages multicollinearity among predictors and facilitates the creation of sparse models through L1 regularization. The LASSO regression model was constructed using the Forward LR method and validated using 10-fold cross-validation. This approach was preferred over alternative methods, such as stepwise regression, which tend to overfit, especially in scenarios involving high-dimensional data sets. Ten-fold cross-validation was instrumental in optimizing the penalty parameter (λ), ensuring model generalizability. Additional statistical analyses included univariate and multivariate logistic regression, along with the generation of receiver operating characteristic (ROC) curves and calibration plots. All analyses adhered to a significance threshold set at *P* ≤ 0.05.

## 3 Results

### 3.1 General patient information

**FIGURE 1:**
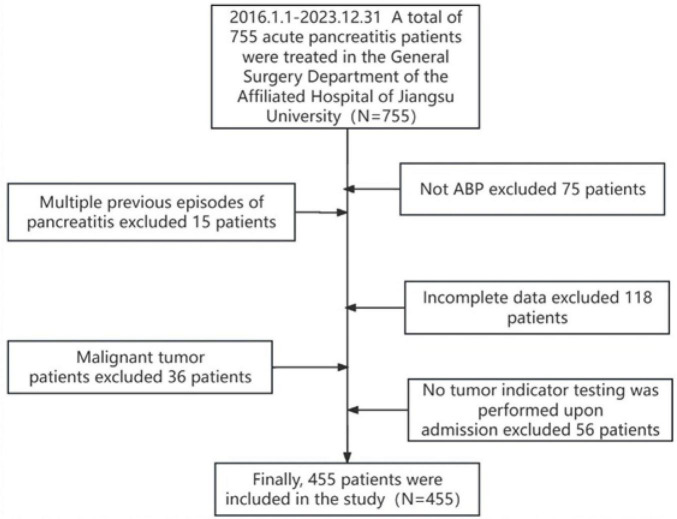
Patient screening flowchart.

In this study, 755 patients who met the diagnostic criteria for ABP were initially identified. After applying the inclusion and exclusion criteria, 455 patients were selected for the final analysis (Figure 1). The final cohort consisted of 396 patients in the MAP group, with a mean age of 63.96 ± 16.17 years; 38 in the MSAP group, with a mean age of 65.74 ± 15.71 years; and 21 in the SAP group, with a mean age of 64.71 ± 17.36 years. Analysis of demographic data revealed no significant differences in age, body mass index (BMI), sex distribution, prevalence of hypertension, or incidence of diabetes mellitus across the three groups (all *P* > 0.05). Similarly, admission laboratory values, including blood amylase, blood lipase, and urinary amylase levels, showed no significant intergroup differences (all *P* > 0.05). However, hospitalization duration differed significantly among the groups (*P* < 0.05), specifically 32.10 ± 10.75 days in the SAP group, 25.47 ± 11.67 days in the MSAP group, and 12.30 ± 5.09 days in the MAP group. Notably, there were no patient deaths during the study period. Complete demographic and clinical data for the cohort are detailed in Table 1.

**TABLE 1:** General information of patients.

Characteristics	Pathological type			χ^2^/*F*	*P*
	**MAP (*n* = 396)**	**MSAP (*n* = 38)**	**SAP (*n* = 21)**		
Age (years)	63.96 ± 16.17	65.74 ± 15.71	64.71 ± 17.36	0.2214	0.8015
BMI	27.44 ± 5.69	27.26 ± 4.86	29.13 ± 4.91	0.9449	0.3895
Sex (N,%)				2.730	0.2554
Male	190 (47.98)	22 (57.89)	13 (61.90)		
Female	206 (52.05)	16 (42.11)	8 (38.10)		
Hypertension, N(%)				1.799	0.4069
Yes	165 (41.67)	19 (50.00)	11 (52.38)		
No	231 (58.33)	19 (50.00)	10 (47.62)		
Diabetes, N(%)				0.2016	0.9041
Yes	50 (12.63)	4 (10.53)	3 (14.29)		
No	346 (87.37)	34 (89.47)	18 (85.71)		
Serum amylase (U/L)	784.5 ± 948.2	1066 ± 914	993.2 ± 737.1	1.932	0.1460
Serum lipase (U/L)	1709 ± 867.1	1963 ± 706.6	1938 ± 675.6	2.148	0.1179
Urinary amylase (U/L)	4490 ± 1956	3896 ± 2210	3867 ± 1712	2.418	0.0902
Length of hospitalization (days)	12.30 ± 5.09	25.47 ± 11.67	32.10 ± 10.75	167.0	< 0.001

### 3.3 Comparison of inflammatory and tumor indicators in different types of pancreatitis

The comparative analysis of admission laboratory values indicated distinct expression patterns of inflammatory and tumor markers across the severity groups. There were no statistically significant differences in alpha-fetoprotein (AFP), carcinoembryonic antigen (CEA), or carbohydrate antigen CA724 levels among the three groups (all *P* > 0.05) (Table 2). However, significant differences were observed for carbohydrate antigen 50 (CA50), CA19-9, CA125, CA242, ferritin levels, WBC count, hs-CRP, total bilirubin, direct bilirubin, alanine aminotransferase, and aspartate aminotransferase across the MAP, MSAP, and SAP groups (all *P* < 0.05) (Table 2).

**TABLE 2:** Comparison of inflammatory and tumor indicators in different types of pancreatitis.

Inflammatory and tumor indicators	Pathological type			χ^2^/*F*	*P*
	**MAP (*n* = 396)**	**MSAP (*n* = 38)**	**SAP (*n* = 21)**		
AFP (ng/mL)	2.67 ± 1.61	2.56 ± 1.926	3.16 ± 3.29	0.8961	0.4089
CEA (ng/mL)	2.64 ± 3.44	3.78 ± 5.39	2.72 ± 1.44	1.721	0.1800
CA50 (U/mL)	36.7 ± 72.3	70.07 ± 100.8	121.9 ± 159	13.31	<0.001
CA19-9 (U/mL)	83.94 ± 199.5	228.2 ± 367.6	234.0 ± 241.3	11.35	<0.001
CA125 (U/mL)	24.45 ± 30.6	51.78 ± 77.95	45.11 ± 41.54	11.72	<0.001
CA724 (ng/mL)	2.225 ± 3.917	2.207 ± 2.240	1.984 ± 1.863	0.04152	0.9593
CA242 (IU/mL)	8.139 ± 14.94	13.82 ± 20.22	13.21 ± 16.58	3.197	0.0418
Ferritin (ng/mL)	567.9 ± 520.1	792.5 ± 613.9	776.4 ± 588.4	4.358	0.0133
WBC (×10^9^)	9.622 ± 4.19	11.28 ± 4.79	11.99 ± 5.37	5.256	0.0055
Hs-CRP (mg/L)	38.70 ± 46.09	61.75 ± 61.11	67.36 ± 51.63	7.157	0.0009
Total bilirubin (μmol/L)	33.45 ± 32.94	57.04 ± 53.75	50.63 ± 37.44	9.568	< 0.001
Direct bilirubin (μmol/L)	17.91 ± 24.72	35.94 ± 44.82	31.65 ± 30.78	9.610	< 0.001
Alanine aminotransferase (U/L)	148.3 ± 173.6	223.9 ± 192.1	250.6 ± 270.3	5.857	0.0031
Aspartate aminotransferase (U/L)	129.2 ± 173.5	220.5 ± 253.1	283.4 ± 363.4	9.591	< 0.001

### 3.4 Construction of LASSO regression prediction models

**FIGURE 2:**
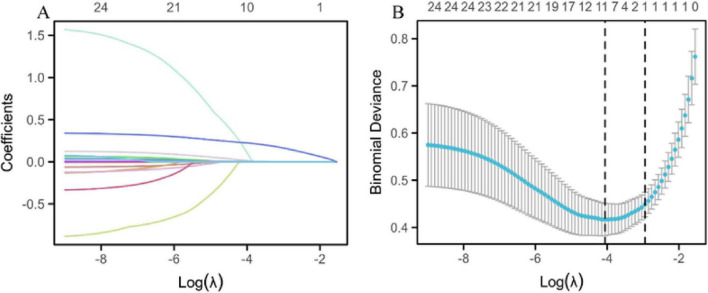
LASSO regression prediction model. **(A)** LASSO regression variable trajectories. The horizontal axis represents the logarithmic value of lambda [log(λ)], and the vertical axis represents the coefficient values of the variables. The upper horizontal axis shows the number of non-zero coefficient variables in the model, and each curve represents the trajectory of the coefficient of an individual variable. **(B)** Log(λ) values and model errors. The lower x-axis represents the log(λ) values, and the upper x-axis indicates the number of non-zero coefficient variables corresponding to each λ value. The y-axis represents the classification error rate (Class) or the -2 log-likelihood function value (Deviance) of the model under different metrics. Dots represent the mean likelihood deviation corresponding to each λ during the cross-validation process. Vertical lines (error bars) represent the standard error of the likelihood deviation at each λ. The left dashed line indicates the optimal lambda value (lambda.min), while the right dashed line indicates the lambda value (lambda.1se) within one standard error of the minimum.

A LASSO regression prediction model was developed to assess disease severity, using MAP status as the binary dependent variable. The model used LASSO regression to screen variables, ensuring the selection of the most relevant predictors while minimizing overfitting. This was followed by validation using 10-fold cross-validation to enhance the reliability of the model’s predictions. After the LASSO regression screening process, the variables selected as significant predictors included sex, WBC count, hs-CRP, CA50, CA19-9, CA125, urinary amylase, total bilirubin, aspartate aminotransferase, and hospitalization duration (Figure 2).

### 3.5 Logistic regression analysis and ROC curves for variables

**FIGURE 3:**
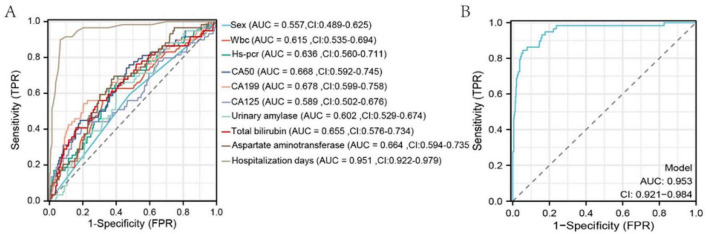
ROC curves of selected variables and the overall model after LASSO regression. The x-axis represents 1-specificity; the closer to zero, the higher the specificity. The y-axis represents sensitivity; the higher the value, the better the accuracy.

Univariate and multivariate logistic regression analyses were conducted using variables selected by LASSO regression. Univariate analysis identified significant risk factors for MSAP or SAP (all *P* < 0.05): WBC count (*P* = 0.002, 95% CI: 1.035–1.166), hs-CRP (*P* < 0.001, 95% CI: 1.004–1.014), CA50 (*P* < 0.001, 95% CI: 1.003–1.007), CA19-9 (*P* < 0.001, 95% CI: 1.001–1.003), CA125 (*P* < 0.001, 95% CI: 1.006–1.017), urinary amylase (*P* = 0.029, 95% CI: 1.000–1.000), total bilirubin (*P* < 0.001, 95% CI: 1.006–1.019), aspartate aminotransferase (*P* < 0.001, 95% CI: 1.001–1.003), and duration of hospitalization (*P* < 0.001, 95% CI: 1.265–1.463). Multivariate analysis demonstrated that the duration of hospitalization (*P* < 0.001, 95% CI: 1.240–1.437) was an independent risk factor for MSAP or SAP (Table 3). ROC curve analysis showed that all variables had an area under the curve (AUC) values > 0.5, indicating a good predictive value for distinguishing patients from non-MAP (Figure 3A). The overall model exhibited an AUC of 0.953 (95% CI: 0.921–0.984), indicating high accuracy (Figure 3B).

**TABLE 3:** One-way and multi-factor logistic regression analyses of variables selected by LASSO regression brushing.

Characteristics	Total (N)	Univariate analysis		Multivariate analysis	
		**Odds ratio (95% CI)**	***P*-value**	**Odds ratio (95% CI)**	***P*-value**
Sex	455				
0	230	Reference			
1	225	1.581 (0.907–2.756)	0.106		
WBC	455	1.099 (1.035–1.166)	**0.002**	1.080 (0.972–1.199)	0.151
Hs-CRP	455	1.009 (1.004–1.014)	**< 0.001**	1.006 (0.998–1.015)	0.146
CA50	454	1.005 (1.003–1.007)	**< 0.001**	1.002 (0.996–1.008)	0.514
CA19-9	455	1.002 (1.001–1.003)	**< 0.001**	1.000 (0.998–1.002)	0.743
CA125	455	1.012 (1.006–1.017)	**< 0.001**	1.007 (0.998–1.016)	0.125
Urinary amylase	455	1.000 (1.000–1.000)	**0.029**	1.000 (1.000–1.000)	0.089
Total bilirubin	455	1.013 (1.006–1.019)	**< 0.001**	1.008 (0.997–1.019)	0.139
Aspartate aminotransferase	455	1.002 (1.001–1.003)	**< 0.001**	1.001 (0.999–1.003)	0.180
Hospitalization days	455	1.360 (1.265–1.463)	<0.001	1.335 (1.240–1.437)	**< 0.001**

Bold values represent statistically significant values.

### 3.6 Construction of the column chart prediction model and diagnosis of the model Calibration

**FIGURE 4:**
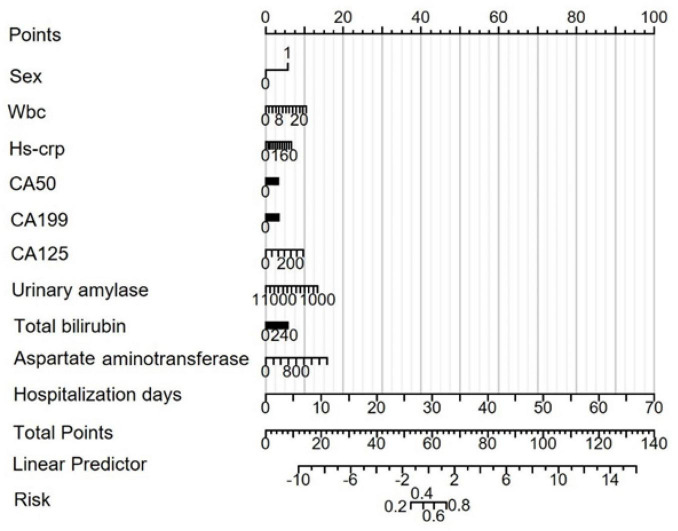
Risk prediction nomogram model for MAP status. Points: Individual scores corresponding to each predictor variable at different values. Total Points: The total score obtained by adding the scores for all variables. Linear Predictor: The linear prediction value based on the total points.

**FIGURE 5:**
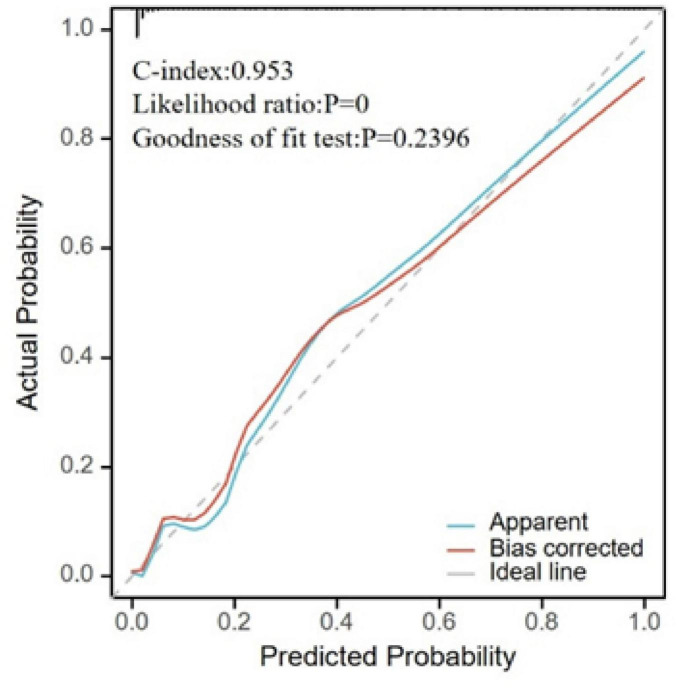
Calibration plot for model validation. The horizontal axis represents the survival probability predicted by the model, and the vertical axis represents the actual observed survival probability. The “Apparent” curve represents the raw predicted curve; the “Bias-corrected” curve represents the calibration curve after adjustment; the “Ideal” line represents perfect calibration.

Based on the results from the LASSO-logistic regression analysis, we developed a risk prediction model for MAP status using the following variables: sex, WBC count, hs-CRP, CA50, CA19-9, CA125, urinary amylase, total bilirubin, aspartate aminotransferase, and duration of hospitalization (Figure 4). Model calibration was assessed through diagnostic validation, which yielded a concordance index (C-index) of 0.953. The likelihood ratio test resulted in a P < 0.001, and the Hosmer–Lemeshow goodness-of-fit test returned a *P* = 0.2396, indicating strong model significance and overall accuracy (Figure 5).

## 4 Discussion

The aim of this study was to explore the clinical significance of elevated tumor markers in patients with biliary pancreatitis by examining clinical phenomena and to propose a clinical prediction model for MAP using machine learning. This model is intended to help clinicians adjust treatment plans.

In this study, our findings demonstrated significantly higher levels of CA50, CA19-9, CA125, CA724, CA242, ferritin, leukocyte count, hs-CRP, total bilirubin, direct bilirubin, alanine aminotransferase, and aspartate aminotransferase in SAP and MSAP cases compared to MAP cases. Although these markers are typically associated with gastrointestinal malignancies such as pancreatic, gastric, and colorectal cancers, recent studies confirm their elevation also occurs in benign conditions (17). For example, Song et al. noted that CA50 levels are elevated in patients with type 2 diabetes and atherosclerosis. Furthermore, trace amounts of CA19-9 are normally present in the epithelial cells of the salivary glands, prostate, pancreas, mammary glands, stomach, bile ducts, gallbladder, and bronchial tubes (18, 19), and elevated levels have been found in patients with conditions like choledocholithiasis and acute cholangitis (20). Notably, patients with AP and elevated CA19-9 face a significantly higher risk of developing pancreatic cancer within 5 years (21). CA125 is also known to be elevated in benign ovarian tumors (22), and slight increases in CA19-9/CA50 and CA19-9/CA242 ratios have been observed in xanthogranulomatous cholecystitis (23). Elevated serum ferritin levels have been reported in patients with hepatitis as well (24). While our study confirms the predictive value of tumor markers for assessing the severity of ABP, it also highlights the need to consider their potential for false positivity in inflammatory conditions. To minimize the impact of false positives, our LASSO regression model incorporated L1 regularization to eliminate multicollinear variables, focusing on markers associated with ABP severity, such as hospitalization duration and hs-CRP. Ten-fold cross-validation was used to optimize the model’s generalizability. The model achieved high specificity (AUC = 0.953); however, future studies should quantify false-positive rates across diverse clinical scenarios and incorporate complication-specific variables, such as findings from endoscopic retrograde cholangiopancreatography (ERCP) and characteristics of choledocholithiasis.

Assessing the severity of AP is crucial for guiding treatment strategies. Various methods are currently employed to predict AP severity. Studies have indicated that the combined sensitivities of the APACHE-II, BISAP, and Ranson scores are 0.67 (95% CI: 0.60–0.73), 0.59 (95% CI: 0.48–0.70), and 0.61 (95% CI: 0.40–0.79), respectively, suggesting that there is significant potential for improvement in their predictive accuracy (25). Additionally, some have utilized CT scans for predicting AP severity (26). In recent years, with advancements in artificial intelligence, several machine learning-based AP prediction models have been developed (27, 28). However, these models still require effective validation to ensure their reliability and accuracy in clinical settings.

In this study, LASSO regression was employed to screen variables with MAP status as the dependent variable, followed by logistic regression analysis on the identified variables. WBC count, hs-CRP, CA50, CA19-9, CA125, urinary amylase, total bilirubin, aspartate aminotransferase, and hospitalization duration were pinpointed as risk factors for MSAP or SAP. Multivariate logistic regression analysis confirmed hospitalization duration as an independent risk factor for MSAP or SAP (Table 3). The significant association between hospitalization duration and disease severity suggests that more severe cases require extended care due to complications and the need for tailored treatment approaches. However, it is important to note that hospitalization length can also be influenced by factors unrelated to disease severity, such as delays in discharge planning or specific treatment protocols, which were not explicitly analyzed in this study. Future studies should include complication-specific variables to elucidate this relationship more clearly. ROC curve analysis demonstrated that all variables had predictive value for distinguishing whether a patient was non-MAP (AUC > 0.5) (Figure 3A). The overall model exhibited high accuracy (AUC = 0.953). A risk prediction nomogram for MAP assessment was developed, and the model’s fit was evaluated using calibration metrics, revealing excellent performance (C-index = 0.953), significant results from the likelihood ratio test (*P* < 0.001), and a good fit (*P* = 0.240). These findings underscore the importance of admission tumor marker levels in the assessment of MAP, highlighting their significant clinical implications for patient management.

While traditional scoring systems offer general severity stratification for AP, our model provides a biliary pancreatitis-specific risk assessment by integrating tumor markers that are elevated during inflammatory states. This specificity allows for the earlier identification of patients at risk for progression to MSAP or SAP, enabling timely clinical interventions such as ERCP or intensive care monitoring. In contrast to conventional approaches, our model incorporates dynamic biomarkers along with hospitalization duration data, capturing the real-time inflammatory status and clinical progression of patients. This methodology addresses specific limitations of existing scoring systems, particularly the delayed predictive capacity of the APACHE-II and the subjective components of the BISAP scoring system. It is important to note that our model is not intended to replace these standard scoring systems but rather to complement them, enhancing risk stratification during the critical early phase of admission when traditional systems may be least effective. This integration offers a more nuanced approach to managing biliary pancreatitis, potentially improving patient outcomes by facilitating earlier and more targeted interventions.

This study is subject to a few limitations. First, the lack of long-term patient follow-up precluded the ability to assess clinical outcomes over time and the potential development of malignancy. Second, our analysis did not consider two potentially significant factors: the presence of concomitant choledocholithiasis and whether patients underwent ERCP procedures, both of which could significantly influence outcomes. Third, the retrospective single-center design spanning from 2016 to 2023 introduces inherent risks of selection bias, including issues such as missing data and the exclusion of patients who did not undergo admission tumor marker testing. Additionally, there may be potential confounding from unmeasured variables such as variations in treatment approaches and differences in physician practice patterns.

Regarding model validation, while our machine learning model demonstrated strong internal accuracy with an AUC of 0.953, its generalizability across diverse populations and healthcare settings has yet to be confirmed due to the lack of external validation. To overcome these limitations, it is crucial to conduct large-scale, prospective multicenter studies that validate the model in diverse populations. Such studies should incorporate real-time data collection to minimize bias. Additionally, external validation is needed, involving the assessment of the model using independent datasets from different hospitals. This step is essential to ensure that the model performs consistently and reliably in various clinical environments.

## 5 Conclusion

The elevation of tumor markers has demonstrated significant clinical importance in managing patients with biliary pancreatitis. We have developed a machine-learning-based clinical prediction model for MAP by effectively screening variables. This model has the potential to assist clinicians in refining their treatment plans, enabling more personalized and timely interventions for patients. This tool not only underscores the significance of tumor markers in predicting disease severity but also highlights the utility of advanced analytical techniques in enhancing patient management strategies.

## Data Availability

The raw data supporting the conclusions of this article will be made available by the authors, without undue reservation.
